# Effects of Bone Marrow-Derived Mesenchymal Stem Cells on the Axonal Outgrowth through Activation of PI3K/AKT Signaling in Primary Cortical Neurons Followed Oxygen-Glucose Deprivation Injury

**DOI:** 10.1371/journal.pone.0078514

**Published:** 2013-11-12

**Authors:** Yong Liu, Yixian Zhang, Longzai Lin, Feifei Lin, Tin Li, Houwei Du, Ronghua Chen, Wei Zheng, Nan Liu

**Affiliations:** 1 Department of Neurology, Fujian Medical University Union Hospital, Fuzhou, Fujian, People Republic of China; 2 Institute of Cerebral Vascular Disease of Fujian Province, Fuzhou, Fujian, People Republic of China; 3 Department of Rehabilitation, Fujian Medical University Union Hospital, Fuzhou, Fujian, People Republic of China; School of Pharmacy, Texas Tech University HSC, United States of America

## Abstract

**Background:**

Transplantation with bone marrow-derived mesenchymal stem cells (BMSCs) improves the survival of neurons and axonal outgrowth after stroke remains undetermined. Here, we investigated whether PI3K/AKT signaling pathway is involved in these therapeutic effects of BMSCs.

**Methodology/Principal Findings:**

(1) BMSCs and cortical neurons were derived from Sprague-Dawley rats. The injured neurons were induced by Oxygen–Glucose Deprivation (OGD), and then were respectively co-cultured for 48 hours with BMSCs at different densities (5×10^3^, 5×10^5^/ml) in transwell co-culture system. The average length of axon and expression of GAP-43 were examined to assess the effect of BMSCs on axonal outgrowth after the damage of neurons induced by OGD. (2) The injured neurons were cultured with a conditioned medium (CM) of BMSCs cultured for 24 hours in neurobasal medium. During the process, we further identified whether PI3K/AKT signaling pathway is involved through the adjunction of LY294002 (a specific phosphatidylinositide-3-kinase (PI3K) inhibitor). Two hours later, the expression of pAKT (phosphorylated AKT) and AKT were analyzed by Western blotting. The length of axons, the expression of GAP-43 and the survival of neurons were measured at 48 hours.

**Results:**

Both BMSCs and CM from BMSCs inreased the axonal length and GAP-43 expression in OGD-injured cortical neurons. There was no difference between the effects of BMSCs of 5×10^5^/ml and of 5×10^3^/ml on axonal outgrowth. Expression of pAKT enhanced significantly at 2 hours and the neuron survival increased at 48 hours after the injured neurons cultured with the CM, respectively. These effects of CM were prevented by inhibitor LY294002.

**Conclusions/Significance:**

BMSCs promote axonal outgrowth and the survival of neurons against the damage from OGD in vitro by the paracrine effects through PI3K/AKT signaling pathway.

## Introduction

As one of potential therapeutic arms, it has been demonstrated that transplantation with bone marrow-derived mesenchymal stem cells (BMSCs) can promote functional recovery and nervous tissue repair in a vast number of previous studies associated with stroke [1], [2], [3]. Several factors may be involved in BMSCs’ therapeutic effects: induction of neurogenesis and angiogenesis, transdifferentiation, neuroprotection, and activation of endogenous neurorestorative processes [3], [4]. In fact, axonal outgrowth and repair in the nervous system underlie functional plasticity and behavioral recovery after ischemic stroke [1]. More recently, it has been widely accepted that BMSCs improve post-stroke functional recovery primarily by its paracrine effects, in turn which promote axonal outgrowth and neuron survival [5], [6], [7], [8], [9]. However, the mechanism remains undetermined.

Phosphatidylinositol-3-kinase (PI3K)/AKT signaling pathways can be activated by a variety of extracellular stimuli and regulate a wide range of cellular processes, including cell motility, cell survival and proliferation, cell cycle progression [10]. Recent studies showed that the activation of PI3K/AKT are involved in cell survival [11] and axonal outgrowth [12] in neurons. Growth associated protein (GAP-43), a neuron-specific protein, dramatically increased during regeneration and development of nervous tissue [12]. GAP-43 is a neurotrophin-dependent membrane bound phosphoprotein found in the growth cone and axon of neurons [13]. A study reports that activation of PI3K/Akt by insulin-like growth factor-1(IGF-1) results in enhanced expression of GAP-43 in dorsal root ganglion (DRG) neurons [14]. Thus, we hypothesized that BMSCs may promote the axonal outgrowth and the neuron survival by paracrine effects through PI3K/AKT signaling pathway, and which was confirmed by a vitro oxygen–glucose deprivation (OGD) model of cerebral ischemia in this study.

## Methods

### Ethics Statement

Animals were cared for in accordance with the National Institute of Health Guide for the Care and Use of Laboratory Animals (NIH Publications No. 80-23) revised 1996. All study procedures were approved by the Fujian Medical University Institutional Animal Care and Use Committee.

### Culture and Differentiation Assay of BMSCs

BMSCs were prepared from tibias and femurs of Sprague–Dawley (60–80 g) male rats as described by our former study [15], [16]. In brief, SD rats were euthanized and bone marrow was harvested. Bone marrow cells were placed into 25 cm^2^ flasks and cultured in a solution of Dulbecco’s Modified Eagle’s Medium (DMEM; Sigma) containing 10% fetal bovine serum and 100 U/ml penicillin/streptomycin, incubated at 37°C in 5% CO_2_. Culture medium was replaced approximately every three days. When cells grew to approximately 80–90% confluence, they were expanded in additional 25 cm^2^ flask. Following the second generation, these cells were trypsinized using trypsin-EDTA 0.05% (Gibco) and administered to differentiation assay.

The differentiation of BMSCs towards the osteogenic and adipogenic lineage was carried out as previously described [17], [18]. In osteoblast differentiation assay, BMSCs were cultured for three weeks in a solution of DMEM containing 10% fetal bovine serum, 10 mM ß-glycerophosphate, 50 µg/ml ascorbic acid, 10^−7^ M dexamethasone (Sigma). In adipocyte differentiation assay, BMSCs were cultured for three weeks in a solution of DMEM containing 10% fetal bovine serum, 10 µg/ml insulin, 10^−6^ M dexamethasone, 100 µg/ml 3-isobutyl-1-methylxantine (Sigma). After three weeks, Von kossa dyes and Oil-red-O dyes were used to identify osteoblasts and adipocytes respectively.

### Primary Cultures of Cortical Neurons

Primary cultures of cortical neurons were prepared from pregnant Sprague-Dawley rats as described with slight modification [19]. Briefly, pregnant 16∼18 days old rats were euthanized and embryos were harvested in sterile conditions. Fetal brains were dissected out by forceps on ice and placed in petri dish containing ice-cold DMEM. Meninges were removed under microscope and cortical tissue were chopped into 1 mm×1 mm×1 mm pieces with micro-spring scissors. These tissues were transferred to 15 ml tubes containing trypsin-EDTA (0.025% in PBS). The tubes were incubated in 37°C chamber for 15 minutes and were agitated every 5 minutes. After stopping the trypsinization with 5 ml 20% FBS, these tissues were triturated 15 times with a Pasteur pipet. Then cell clumps were left to settle for 2 minutes to allow debris to settle and transfer the supernatant to a 15 ml eppendorf tube. The supernatant was filtered through a 75 µm pore-sized filter and centrifuged in a tube for 2 minutes at 1000 r/min. The cells were resuspended in the neurobasal medium (GIBCO) containing 2% B27(GIBCO), and 0.5 mM glutamine, 50 U/ml penicillin/streptomycin. 2×10^5^ and 2×10^6^ cells respectively were seeded on poly-L-lysine-coated 24 mm×24 mm coverslips (0.05 mg/ml) for immunofluorescence and Western, and cultured in chamber (37°C, 5% CO_2_). Culture medium was first replaced after 24 hours, then half of the medium was replaced with fresh medium every three days.

### Oxygen–Glucose Deprivation (OGD)

Oxygen–Glucose Deprivation (OGD) model was established as privously described with slight modification [20]. Primary cortical neurons were cultured from embryonic SD rat embryos (16–18 day). On DIV 5, cells were washed with phosphate-buffered saline and cultured in glucose-free DMEM (GIBCO) after incubated in an anaerobic chamber (95% N2, 5% CO_2_) for 30 min to remove residual oxygen. Then cells were placed in an anaerobic chamber containing 5% CO_2_ and 95% N2 at 37°C for OGD. Cells were returned to original medium 90 min later, and placed in a normoxic chamber (37°C, 5% CO_2_).

### Coculture of Post-OGD Neurons with BMSCs

**Figure 1:**
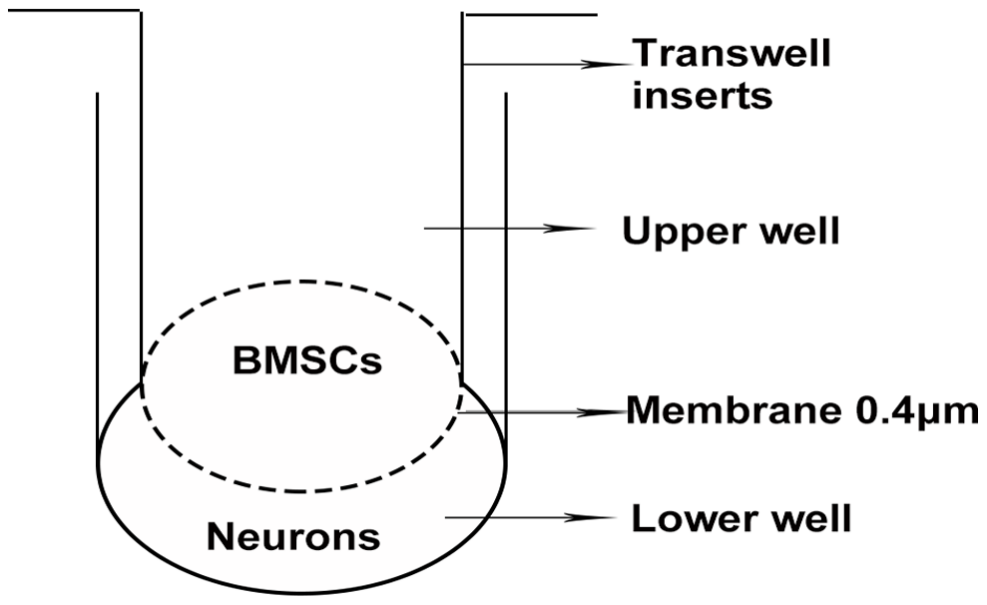
Transwell system. Transwell system is used to coculture BMSCs and neurons. Nutritive substance can freely penetrate the membrane, but cells can not pass through the membrane.

To confirm whether BMSCs promote axonal outgrowth by paracrine effects, a Transwell co-culture system was used (Fig. 1). The 3^rd^ passage of BMSCs at different densities 5×10^3^ and 5×10^5^/ml were seeded to the upper well of the six-well Transwell system and cultured for two days respectively. After that, the medium were changed with 1 ml neurobasal medium. Cortical neurons were harvested and cultured for five days, then the cell injury was induced by OGD. Ninety minutes later, neurons were immediately placed into the lower well of the six-well Transwell system and 1 ml neurobasal medium added. Thus the per well of the six-well Transwell system include 2 ml neurobasal medium total. Fourty-eight hours later, the length of axonal outgrowth and the expression of GAP-43 were measured. Cells those cultured in the neurobasal medium were used as control. Four experimental groups: Control group (normal culture), OGD group, OGD+5×10^3^ BMSCs group, OGD+5×10^5^ BMSCs group.

### Preparation of BMSCs Conditioned Medium (CM)

The 3^rd^ passage of cultured BMSCs at a density of 3×10^4^ to 5×10^4/^ml were cultured for three days. The culture medium was replaced with a volume of 1 ml neurobasal medium. Total conditioned medium (CM) were collected and debris was removed by rinsing through 0.2 µm filter system after 24 hours later, then stored at −20°C.

### Blocking Experiment of PI3K/AKT

PI3K/AKT signaling pathways regulate a wide range of cellular processes, including cell survival and proliferation. To avoid the possible effects of inhibitor LY294002 on survival and proliferation of BMSCs through PI3K/AKT, CM from BMSCs was applied to culture OGD-injured neurons involved in investigating molecular pathways of neuroprotection of BMSCs as described [11], [21]. In brief, after OGD injured, neurons were cultured in 2 ml CM with or without LY294002 (20 µM). At 2 hours of culture age, the expression of pAKT and AKT were analyzed by Western blotting. The length of axons, the expression of GAP-43 and the survival of neurons were measured at 48 hours. Four experimental groups: OGD group, OGD+CM group, OGD+CM+ LY294002 (20 µM) group, OGD+ LY294002 (20 µM) group.

### Western Blot Analysis

According to the instruction of RIPA (Beyotime) lysis buffer, protein was extracted from primary cortical neurons. The sample was centrifuged at 14,000 r/min for 15 min at 4°C, and the supernatant was collected and used for protein analysis. Protein concentration was determined with the BCA protein assay (Beyotime). 20 µg protein of every sample was loaded onto the 10% SDS gel, separated by electrophoresis and transferred to polyvinylidene fluoride membranes (PVDF, Millipore). Blocking of membranes was carried out with blocking solution (Beyotime) for 2 h at room temperature. Then the membranes were incubated with the following primary antibodies overnight at 4°C: rabbit anti-pAKT monoclonal antibody (1∶1000, Cell signaling Technology), rabbit anti-AKT polyclonal antibody (1∶1000, Cell signaling Technology), rabbit anti-GAP-43 monoclonal antibody (1∶1000, Cell signaling Technology), mouse anti-GAPDH monoclonal antibody (1∶500, Wuhan boster bio-engineering limited company). After washing three times, the membranes were incubated by following secondary antibody for 2 h at room temperature: goat anti-rabbit IgG-HRP (1∶4000, Zhongshan Goldenbridge Biotechnology), goat anti-mouse IgG-HRP (1∶4000, Zhongshan Goldenbridge Biotechnology). Signals on membranes were visualized by an ECL western blotting detection kit (Beyotime) on Kodak XTB-01 films. All experiments were repeated six times.

### Immunofluorescence Staining and Axonal Outgrowth Assay

Culture plates were washed two times with 37°C PBS, and cells were fixed in 4% paraformaldehyde (pH 7.4) for 15 min. Blocking and permeabilization were carried out in 3% normal donkey serum (Jackson Immunoresearch), and 0.1% TritonX-100(sigma). Cells were incubated by following primary antibodies overnight at 4°C: rabbit anti-GAP-43 monoclonal antibody (1∶200, Cell signaling Technology), mouse anti-class III β-Tubulin monoclonal antibody (1∶200, Beyotime). After washing three times, the following secondary antibodies were incubated for 2 h at room temperature, Cy3 donkey anti-mouse IgG (1∶400, Jackson Immunoresearch), Dylight488 donkey anti-rabbit IgG (1∶400, Jackson Immunoresearch). After washing with PBS, cells were counterstained with Hoechst33342 (sigma) before mounting. Samples were examined under a ZEISS LSM 710 confocal microscope (Germany), and the length of axonal outgrowth was measured with ZEN2009 software (Carl ZEISS). For each group and experiment, 3 visual fields in every coverslip were observed and all experiments were repeated three times.

### Flow Cytometry using Annexin V/PI Staining

For the quantitative assessment of neuronal survival, detection of survival by flow cytometry was performed using the Annexin-V-FLUOS Staining Kit (Roche, Germany). In brief, cortical neurons were seeded in 25 CM^2^ plates and subjected to various treatments as described earlier experimental groups and design. The Annexin V/PI staining was performed according to the manufacturer’s instruction. The neurons were harvested and washed with 4°C PBS three times. Neurons were resuspended by 500 µL incubation buffer. Then 10 µl Annexin- V-FLUOS labeling reagent and 10 µl PI were added into the cell suspension for staining 15 min at room temperature in the dark. Neurons were analyzed immediately using flow cytometry. Survival cells are negative for both Annexin V and PI.

### Statistical Analysis

All data were expressed as mean ± SD. Statistical analysis was evaluated with SPSS13.0 software. One way analysis of variance (ANOVA) followed by Tukey’s multiple comparisons test was used to measure statistical significance. Statistical significance was accepted at P<0.05.

## Result

### Differentiation Capacity of BMSCs

**Figure 2:**
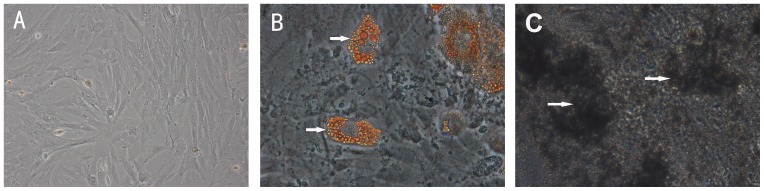
Differentiated capacity of BMSCs. (A) 200× The third passage of BMSCs were fibroblast-like cells and uniformly distributied on the bottom of a plastic flask. (B) 400× Cells stained with Oil-red-O dyes show that BMSCs differentiated into lipid laden adipocyte (red). (C) 400× Cell stained with Von kossa dyes show that BMSCs differentiated into osteocyte of calcium deposits (black).

Initially, only hematopoietic cells can be observed in the harvested marrow cells. After replacing the medium at the third day of culture age, we observed decrease in hematopoietic stem cell lineages and the appearance of spindle-shaped morphology of marrow cells that adhered to the plastic culture flask. The third passage BMSCs were uniformly distributied and adhered to the bottom of flask (Fig. 2A). After three weeks of culture with adipogenic induction and oesteogenic induction, these cells were found differentiated into adipocyte (Fig. 2B) and osteocyte (Fig. 2C).

### Morphology of Primary Rat Cortical Neurons and OGD Injury

**Figure 3:**
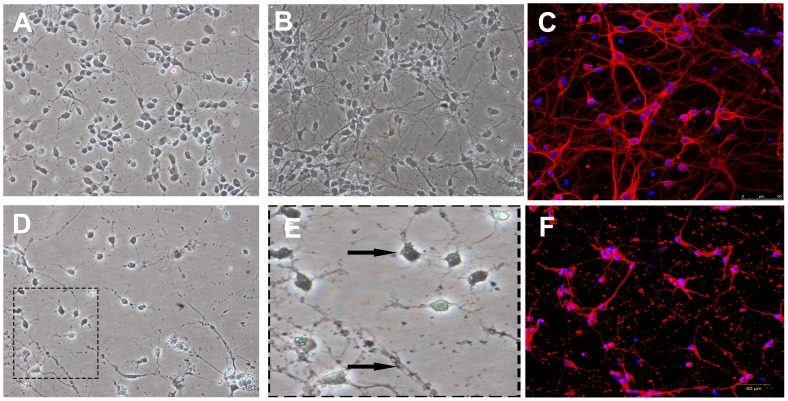
Morphology of primary rat cortical neurons and OGD injury. (A) 200× First day in vitro, neurons were small and adhered to bottom of the flask to grow, with a round body and small neurite. (B) 200× Fifth day in vitro, these neurites of neurons formed an extensive network. (C) Immunofluorescence shows cell body and neurite were labeled by anti-class III β-Tubulin antibody (red), while nuclei were stained with Hoechst33342 (blue). Scale bar = 50 µm. (D) 200× After 24 h, neurite of neurons following by 90 min of OGD injury were degraded and disappeared, with partial dead cell. (arrowhead shown in Fig. E) (magnification). (F) Immunofluorescence shows cell body and neurite after OGD injury. Scale bar = 50 µm.

The typical morphology of cortical neurons growing in coverslip is shown in Fig. 3. After 24 h of culture, neurons which adhered to the coverslip were round and relatively small, with a small neurite outgrowth (Fig. 3A). At five days of culture age, the neurite of neurons was lengthened and formed an extensive network (Fig. 3B). Neurons were stained with anti-class III β-Tubulin antibody by immunofluorescence (Fig. 3C). After OGD injury, we can observe that neurite of neurons were injured and partially disappeared (Fig. 3D,3E,3F).

### BMSCs Enhance Axonal Length of OGD-injured Neurons

**Figure 4:**
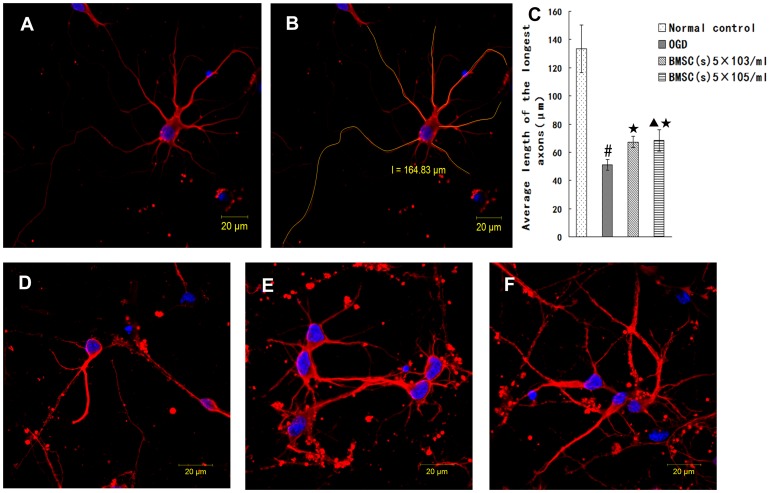
BMSCs treatment promoted axonal outgrowth. (A) Immunofluorescence showing body and neurites of cell (red). (B) Manually traceing morphology of neurites by the ZEN2009 software (yellow). (C) Quantification of the longest axonal length of neurons. Data are expressed as mean±SD. ^#^P<0.01 vs Normal control; ^★^P<0.05 vs OGD; ^▴^P>0.05 vs BMSCs (5×10^3^/ml). (D) OGD (E) OGD+BMSCs (5×10^3^/ml), (F) OGD+BMSCs (5×10^5^/ml). Scale bar = 20 µm.

**Figure 5:**
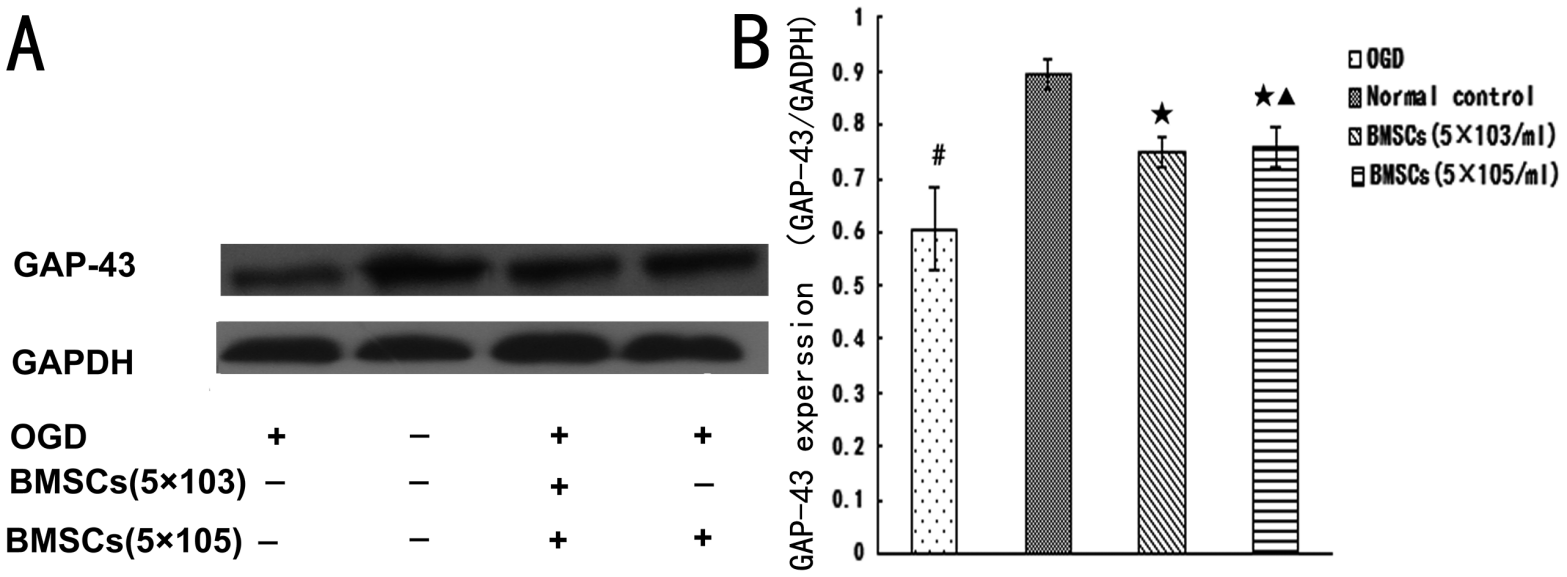
Detection of GAP-43 expression. (A) The most representative image of wesrernblot analysis for GAP-43 expression (B) Statistical graph of GAP-43 expression in different group (n = 6) ^#^P<0.05 vs Normal control; ^★^P<0.05 vs OGD; ^▴^P>0.05 vs BMSCs (5×10^3^/ml).

To investigate the effect of BMSCs on the axonal outgrowth by the secretion, we first investigate whether BMSCs cocultured with OGD-injured neurons by non-contact Transwell system promote axonal outgrowth in vitro. BMSCs were co-cultured for 48 hours with neurons following OGD injury. Immunofluorescent staining for βIII-tubulin identify cell bodies and neurites. Images were captured by ZEISS LSM 710 confocal microscope (Fig. 4A), and the morphology of neurites was manually traced with ZEN2009 software (Carl ZEISS) (Fig. 4B). The length of the longest neurite per neuron was counted. It is shown that, the average length of axon (51.11±3.96 µm) was shortened in OGD group (Fig. 4D) (p<0.01) when compared with Normal control group (Fig. 4A) (133.50±16.92 µm). Compared with OGD group, the average length of axon in OGD+5×10^3^ BMSCs group (67.21±3.97 µm) (Fig. 4E) and OGD+5×10^5^ BMSCs group (68.27±7.61 µm) (Fig. 4F) increased (p<0.05), but there were no significant difference between OGD+5×10^3^ BMSCs group and OGD+5×10^5^ BMSCs group. (p>0.05) (Fig. 4). In comparison with Normal control group (0.89±0.03), the expression of GAP-43 (0.60±0.08) decreased in OGD group (p<0.05). Compared with OGD group, the expression of GAP-43 in OGD+5×10^3^ BMSCs group (0.75±0.03) and OGD+5×10^5^ BMSCs group (0.76±0.04) significantly increased, there were no significant difference between OGD+5×10^3^ BMSCs group and OGD+5×10^5^ BMSCs group (p>0.05) (Fig. 5). These data suggested that BMSCs cocultured with OGD-injured neurons could promote axonal outgrowth and upregulate the expression of GAP-43 in vitro, and the effect of the BMSCs in different density on neurons has no difference.

### BMSCs Conditioned Medium (CM) Improve Axonal Length, Expression of pAKT and GAP-43 of Injured Neurons

**Figure 6:**
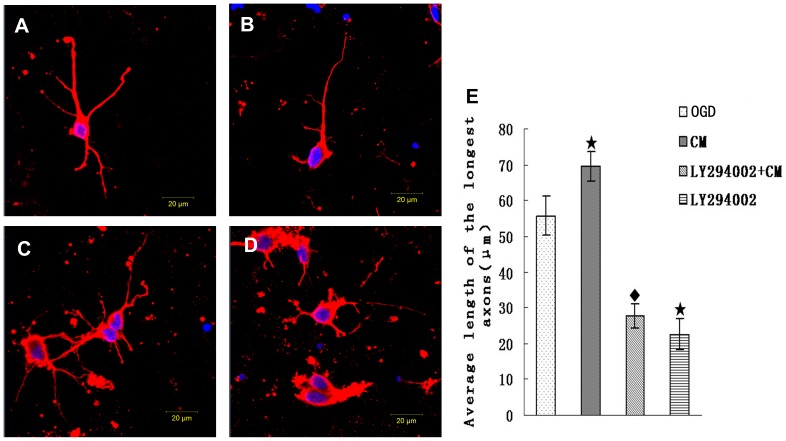
CM treatment promote axonal outgrowth. (A)OGD group: After OGD culture, axons of neurons were injured and shortened. (B)CM group: OGD-Injured axons of neurons were protected and enhanced by CM. (C) LY294002+CM group: Effect of promoting axonal outgrowth of CM was blocked by inhibitor LY294002. (D) LY294002 group. ^★^P<0.05 vs control group; ^♦^P<0.05 vs CM group. Scale bar = 20 µm.

**Figure 7:**
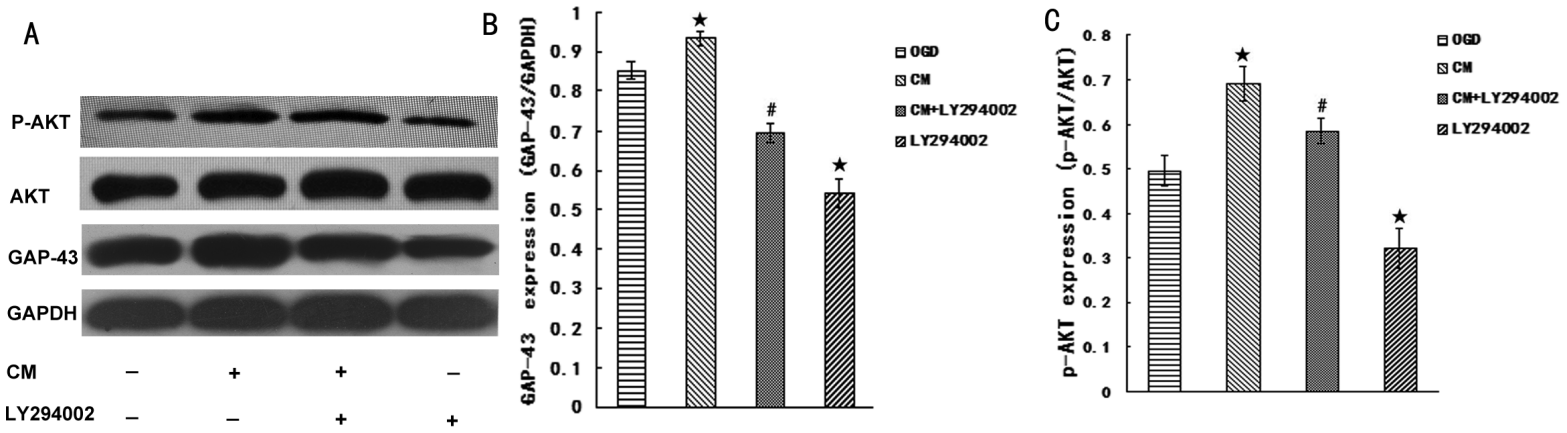
Detection of GAP-43 and p-AKT expression. (A) The most representative image of wesrernblot analysis for GAP-43 and p-AKT expression. (B) Statistical graph of GAP-43 expression in different group (n = 6). (C) Statistical graph of p-AKT expression in different group (n = 6). ^★^P<0.05 vs Control group, ^#^P<0.05 vs CM group.

In order to further investigate the indirect effect of BMSCs on neurons and involved mechanism of signal pathway, OGD-injured neurons were cultured with BMSCs conditioned medium (CM) containing or not containing inhibitor LY294002. As shown (Fig. 6), compared respectively with OGD (55.73±5.47 µm), the average length of axon was decreased in OGD+LY294002 (22.66±4.3 µm) (p<0.05), while the average length of axon was increased in OGD+CM (69.41±4.10 µm) (p<0.05). In comparison with OGD+CM, the average length of axon were decreased (p<0.05) in OGD+CM+LY294002 group (27.69±3.44 µm). The expression of GAP-43 and p-AKT were detected by western blot (Fig. 7). Compared with OGD group, the expression of GAP-43 and p-AKT decreased in OGD+LY294002 group (p<0.05) and increased (p<0.05) in OGD+CM group. Compared with OGD+CM group, the expression of GAP-43 and p-AKT decreased (p<0.05) in OGD+CM+LY294002 group. This data revealed that CM could promote axonal outgrowth and upregulate expression of GAP-43 and p-AKT, the effect was blocked by inhibitor LY294002.

### Localization of GAP-43 Expression at Growing Axons after CM Treatment

**Figure 8:**
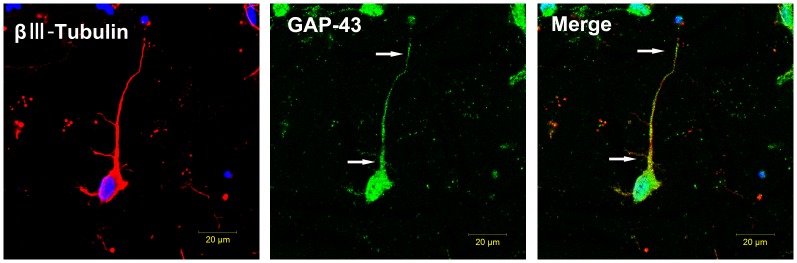
Localization of GAP-43 expression. The left panel shows neuronal marker class III -βTubulin (red) and nucleus (blue). The middle panel shows expression of GAP-43(green), which is expressed in the body and axon. The right panel shows GAP-43 primarily distributes to the proximal and distal axon (white arrows).

Immunofluorescence staining was used to detect the localization of GAP-43 expression at growing axons after CM treatment (Fig. 8). We observed that immunoreactive GAP-43 expressed in the cell body and axon, but GAP-43 staining was intense in the proximal and distal axon, thus indirectly suggesting that the axon may be growing actively.

### Effect of CM on Survival of OGD-injured Neurons

**Figure 9:**
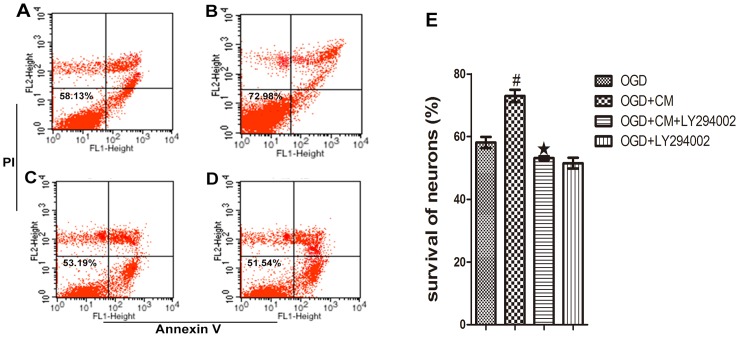
Detection of neuronal survival by flow cytometry. After 48-plot graph. (A) OGD, (B) OGD+CM, (C) OGD+CM+LY294002, (D) OGD+LY294002. (E) ^#^P<0.01 vs OGD, ^★^P<0.01 vs OGD+CM.

To further investigate the effect of BMSCs on survival of OGD-injured neurons and involved in mechanism of signal pathway, OGD-injured neurons were cultured with BMSCs CM containing or not containing inhibitor LY294002. As shown (Fig. 9), compared with the OGD group, CM induced a higher survival of neurons followed OGD injury after 48 h (p<0.01). In addition, CM containing inhibitor LY294002 led to a lower survival compared with the CM group (p<0.01).

## Discussion

Stroke is one of the primary causes of long-term functional disability and death [1], [22]. It has been demonstrated that BMSCs can promote axonal outgrowth, neuronal survival and nerve tissue regeneration by secretory action to support the injured neurons in a vast number of previous studies [5], [23], [24], [25]. In this report, we have shown that BMSCs promoted survival and axonal outgrowth of OGD-injured neurons by paracrine effects. Furthermore, this neuroprotective effect of BMSCs on damage neuron was partially abolished by inhibitor LY294002 of PI3K/AKT signaling pathway.

BMSCs are capable of differentiation into different cell lineage, such as osteoblasts, adipocytes and neuron-like cells [17], [26], [27]. In our study, BMSCs were successfully differentiated into osteoblasts and adipocytes, which show the properties of stem cells in line with our previous study results [15]. This result indicated that cells have been obtained from rats was multipotential BMSCs. BMSCs have become therapeutic cells in stroke diseases because they are easily available and can be rapidly expanded in vitro for autologous transplantation. It has been well known that axonal remodeling is critical to brain repair and function recovery after stroke [28]. In the past decade, several groups found that BMSCs can promote axonal outgrowth in vivo after stroke and also axonal outgrowth of normal dorsal root ganglion (DRG) neurons in vitro [25], [29], [30], [31]. However, the direct evidence that BMSCs promote axonal outgrowth of the injured neurons remains little.

In this study, we found that the axonal length significantly increased when the injured neurons were cultured in the CM from BMSCs or cocultured with BMSCs in non-contact co-cultured system. This indicated that BMSCs promote axonal outgrowth through the paracrine effects, rather than by transdifferentiating into nerve cells. Furthermore, we have found that the effect of BMSCs promoting axonal outgrowth seemed to be not associated with a certain of cell density, which be confirmed by the injured neurons co-cultured with BMSCs at two different densities, i.e., 5×10^3^ cells/ml and 5×10^5^ cells/ml.

Gap-43 as a neuron-specific protein is implicated in axonal growth, plasticity, neuronal differentiation and regeneration [32], [33]. Its activities and distribution are regulated by its dynamic interactions with various neuronal proteins [34]. For instance, axon poor regeneration is partly attributed to inhibitors of the protein Nogo-A. Increased GAP-43 expression may be correlated with Nogo-A inhibition after traumatic brain injury in rats [35]. It has been revealed in a recent study that the axonal outgrowth guidance cue netrin-1 depended on GAP-43 for its function in neurite growth and guidance [36]. In this study, protein expression of growth associated protein (GAP-43) was increased by the treatment of CM or BMSCs in cortical neurons followed OGD injury. Our results have shown that GAP-43 located in growing axon and body, in line with the previous study [32]. Thus,BMSCs can directly promote axonal outgrowth of the injured neurons by paracrine effects though we can not determined the special neuroprotective factors secreted by BMSCs.

To further investigate the mechanism, LY294002, a specific inhibitor of PI3K/AKT signaling pathway was used. PI3K/AKT signaling pathway plays a crucial role in a wide range of cellular processes, including cell cycle progression, cell growth, cell motility, cell adhesion and cell survival [10]. Activation of PI3K/AKT by many different cytokines is beneficial to the regulation of neurite outgrowth and neuronal survival. Insulin-like growth factor-1 (IGF-1) is a neurotrophicfactor and plays a critical role in regulation of membrane expansion at the nerve growth cone by activation of PI3K/AKT [37]. A previous study shows that activation of PI3K/AKT by IGF-1 promoted GAP-43 expression in neurons with excitotoxicity induced by glutamate in vitro [14]. Virdee et al found that nerve growth factor (NGF) stimulation induced a rapid increase in AKT activity which was correlated with sympathetic neuronal survival [38]. Besides, Ma et al recently report that low frequency magnetic stimulation play a role in regulating structural synaptic plasticity of hippocampal neurons via the activation of brain-derived neurotrophic factor (BDNF) and tropomyosin-related kinase B (TrkB) pathways (BDNF-TrkB), including PI3K/AKT signaling pathways [39]. In this study, to avoid possible effects of inhibitor LY294002 on survival and proliferation of BMSCs through PI3K/AKT signaling pathway, just CM from BMSCs was used to culture OGD-injured neurons involved in investigating molecular pathways of neuroprotection of BMSCs. Our results have shown that pAKT expression and axonal outgrowth were increased in OGD-injured neurons cultured with CM. In addition, this study also discovered that CM protected neurons from death induced by OGD, in line with the previous study in vitro [23]. These effects were partially inhibited by inhibitor LY294002. So this study not only demonstrates that the activation of PI3K/AKT is essential for axonal outgrowth of OGD-injured neurons cultured with CM but also supports neuroprotective effects of BMSCs on survival of OGD-injured neurons through PI3K/AKT pathway.

In conclusion, our results support the concept that BMSCs promote axonal outgrowth and the survival of neurons against the damage from OGD in vitro by the paracrine effects through PI3K/AKT signaling pathway. Meanwhile, there are some limitations in our study: First, we can not confirm whether BMSCs of higher or lower density purpose similar action promoting axonal outgrowth and survival of the injured neurons due to just two different densities used in our study. Second, which neurotrophicfactors are beneficial to axonal outgrowth and neuron survival is undetermined. Finally, the downstream molecule mechanism of PI3K/AKT in promoting axonal outgrowth and neuron survival is unclear. Nevertheless, the results from this study should be benefit to extend our understanding for the neuroprotective effects of BMSCs.

## References

[1] Liu Z, Li Y, Zhang ZG, Cui X, Cui Y, et al (2010) Bone marrow stromal cells enhance inter- and intracortical axonal connections after ischemic stroke in adult rats. Journal of Cerebral Blood Flow & Metabolism 30: 1288–1295. 20125183 10.1038/jcbfm.2010.8 PMC2896436

[2] Wei L, Fraser JL, Lu ZY, Hu X, Yu SP (2012) Transplantation of hypoxia preconditioned bone marrow mesenchymal stem cells enhances angiogenesis and neurogenesis after cerebral ischemia in rats. Neurobiol Dis 46: 635–645. 22426403 10.1016/j.nbd.2012.03.002 PMC3353023

[3] Dharmasaroja P (2009) Bone marrow-derived mesenchymal stem cells for the treatment of ischemic stroke. J Clin Neurosci 16: 12–20. 19017556 10.1016/j.jocn.2008.05.006

[4] Kocsis JD, Honmou O (2012) Bone marrow stem cells in experimental stroke. Prog Brain Res 201: 79–98. 23186711 10.1016/B978-0-444-59544-7.00005-6

[5] Crigler L, Robey RC, Asawachaicharn A, Gaupp D, Phinney DG (2006) Human mesenchymal stem cell subpopulations express a variety of neuro-regulatory molecules and promote neuronal cell survival and neuritogenesis. Experimental Neurology 198: 54–64. 16336965 10.1016/j.expneurol.2005.10.029

[6] Scheibe F, Klein O, Klose J, Priller J (2012) Mesenchymal Stromal Cells Rescue Cortical Neurons from Apoptotic Cell Death in an In Vitro Model of Cerebral Ischemia. Cellular and Molecular Neurobiology 32: 567–576. 22290155 10.1007/s10571-012-9798-2 PMC11498621

[7] Wagner W, Roderburg C, Wein F, Diehlmann A, Frankhauser M, et al (2007) Molecular and secretory profiles of human mesenchymal stromal cells and their abilities to maintain primitive hematopoietic progenitors. Stem Cells 25: 2638–2647. 17615262 10.1634/stemcells.2007-0280

[8] Montzka K, Fuhrmann T, Muller-Ehmsen J, Woltje M, Brook GA (2010) Growth factor and cytokine expression of human mesenchymal stromal cells is not altered in an in vitro model of tissue damage. Cytotherapy 12: 870–880. 20662610 10.3109/14653249.2010.501789

[9] Ramos-Cabrer P, Justicia C, Wiedermann D, Hoehn M (2010) Stem cell mediation of functional recovery after stroke in the rat. PLoS One 5: e12779. 20877642 10.1371/journal.pone.0012779 PMC2943902

[10] Cantrell DA (2001) Phosphoinositide 3-kinase signalling pathways. J Cell Sci 114: 1439–1445. 11282020 10.1242/jcs.114.8.1439

[11] Isele NB, Lee HS, Landshamer S, Straube A, Padovan CS, et al (2007) Bone marrow stromal cells mediate protection through stimulation of PI3-K/Akt and MAPK signaling in neurons. Neurochem Int 50: 243–250. 17050038 10.1016/j.neuint.2006.08.007

[12] Choi D-H, Lee K-H, Kim J-H, Kim MY, Lim JH, et al (2012) Effect of 710 nm visible light irradiation on neurite outgrowth in primary rat cortical neurons following ischemic insult. Biochemical and Biophysical Research Communications 422: 274–279. 22580279 10.1016/j.bbrc.2012.04.147

[13] Tedeschi A, Nguyen T, Puttagunta R, Gaub P, Di Giovanni S (2008) A p53-CBP/p300 transcription module is required for GAP-43 expression, axon outgrowth, and regeneration. Cell Death and Differentiation 16: 543–554. 19057620 10.1038/cdd.2008.175

[14] Liu Z, Cai H, Zhang P, Li H, Liu H, et al (2012) Activation of ERK1/2 and PI3K/Akt by IGF-1 on GAP-43 expression in DRG neurons with excitotoxicity induced by glutamate in vitro. Cell Mol Neurobiol 32: 191–200. 21822733 10.1007/s10571-011-9746-6 PMC11498431

[15] Liu N, Zhang Y, Fan L, Yuan M, Du H, et al (2011) Effects of transplantation with bone marrow-derived mesenchymal stem cells modified by Survivin on experimental stroke in rats. Journal of Translational Medicine 9: 105. 21733181 10.1186/1479-5876-9-105 PMC3146839

[16] Soleimani M, Nadri S (2009) A protocol for isolation and culture of mesenchymal stem cells from mouse bone marrow. Nat Protoc 4: 102–106. 19131962 10.1038/nprot.2008.221

[17] Pittenger MF, Mackay AM, Beck SC, Jaiswal RK, Douglas R, et al (1999) Multilineage potential of adult human mesenchymal stem cells. Science 284: 143–147. 10102814 10.1126/science.284.5411.143

[18] Krampera M, Pasini A, Rigo A, Scupoli MT, Tecchio C, et al (2005) HB-EGF/HER-1 signaling in bone marrow mesenchymal stem cells: inducing cell expansion and reversibly preventing multilineage differentiation. Blood 106: 59–66. 15755902 10.1182/blood-2004-09-3645

[19] Wetzel M, Li L, Harms KM, Roitbak T, Ventura PB, et al (2008) Tissue inhibitor of metalloproteinases-3 facilitates Fas-mediated neuronal cell death following mild ischemia. Cell Death Differ 15: 143–151. 17962815 10.1038/sj.cdd.4402246

[20] Choi IY, Yan H, Park Y-K, Kim W-K (2009) Sauchinone reduces oxygen-glucose deprivation-evoked neuronal cell death via suppression of intracellular radical production. Archives of Pharmacal Research 32: 1599–1606. 20091274 10.1007/s12272-009-2113-1

[21] Scheibe F, Klein O, Klose J, Priller J (2012) Mesenchymal Stromal Cells Rescue Cortical Neurons from Apoptotic Cell Death in an In Vitro Model of Cerebral Ischemia. Cell Mol Neurobiol. 10.1007/s10571-012-9798-2 PMC11498621 22290155

[22] Liu N, Huang H, Lin F, Chen A, Zhang Y, et al (2011) Effects of treadmill exercise on the expression of netrin-1 and its receptors in rat brain after cerebral ischemia. Neuroscience 194: 349–358. 21820492 10.1016/j.neuroscience.2011.07.037

[23] Wilkins A, Kemp K, Ginty M, Hares K, Mallam E, et al. (2009) Human bone marrow-derived mesenchymal stem cells secrete brain-derived neurotrophic factor which promotes neuronal survival in vitro. Stem Cell Res. 10.1016/j.scr.2009.02.006 19411199

[24] Uzun G, Subhani D, Amor S (2010) 0Trophic factors and stem cells for promoting recovery in stroke. J Vasc Interv Neurol 3: 3–12. 22518254 PMC3317290

[25] Gu (2012) Bone mesenchymal stromal cells stimulate neurite outgrowth of spinal neurons by secreting neurotrophic factors. Neurological Research. 10.1179/1743132811Y.0000000068 22333032

[26] Wu X, Li SH, Lou LM, Chen ZR (2012) The Effect of the Microgravity Rotating Culture System on the Chondrogenic Differentiation of Bone Marrow Mesenchymal Stem Cells. Mol Biotechnol. 10.1007/s12033-012-9568-x 22669584

[27] Liu W, Lu G, Wang B, Ma Z, Li Y (2012) Transfection of BDNF gene promotes bone mesenchymal stem cells to differentiate into neuron-like cells. Zhong Nan Da Xue Xue Bao Yi Xue Ban 37: 441–446. 22659671 10.3969/j.issn.1672-7347.2012.05.002

[28] Ueno Y, Chopp M, Zhang L, Buller B, Liu Z, et al (2012) Axonal Outgrowth and Dendritic Plasticity in the Cortical Peri-Infarct Area After Experimental Stroke. Stroke 43: 2221–2228. 22618383 10.1161/STROKEAHA.111.646224 PMC3404219

[29] Gutierrez-Fernandez M, Rodriguez-Frutos B, Ramos-Cejudo J, Teresa Vallejo-Cremades M, Fuentes B, et al (2013) Effects of intravenous administration of allogenic bone marrow- and adipose tissue-derived mesenchymal stem cells on functional recovery and brain repair markers in experimental ischemic stroke. Stem Cell Res Ther 4: 11. 23356495 10.1186/scrt159 PMC3706777

[30] Song M, Mohamad O, Gu X, Wei L, Yu SP (2012) Restoration of Intracortical and Thalamocortical Circuits after Transplantation of Bone Marrow Mesenchymal Stem Cells into the Ischemic Brain of Mice. Cell Transplant. 10.3727/096368912X657909 23069268

[31] Bao X, Wei J, Feng M, Lu S, Li G, et al (2011) Transplantation of human bone marrow-derived mesenchymal stem cells promotes behavioral recovery and endogenous neurogenesis after cerebral ischemia in rats. Brain Res 1367: 103–113. 20977892 10.1016/j.brainres.2010.10.063

[32] Chakravarthy B, Rashid A, Brown L, Tessier L, Kelly J, et al (2008) Association of Gap-43 (neuromodulin) with microtubule-associated protein MAP-2 in neuronal cells. Biochemical and Biophysical Research Communications 371: 679–683. 18455509 10.1016/j.bbrc.2008.04.119

[33] Dent EW, Meiri KF (1998) Distribution of phosphorylated GAP-43 (neuromodulin) in growth cones directly reflects growth cone behavior. J Neurobiol 35: 287–299. 9622012 10.1002/(sici)1097-4695(19980605)35:3<287::aid-neu6>3.0.co;2-v

[34] Avwenagha O, Campbell G, Bird MM (2003) Distribution of GAP-43, beta-III tubulin and F-actin in developing and regenerating axons and their growth cones in vitro, following neurotrophin treatment. J Neurocytol 32: 1077–1089. 15044840 10.1023/B:NEUR.0000021903.24849.6c

[35] Marklund N, Bareyre FM, Royo NC, Thompson HJ, Mir AK, et al (2007) Cognitive outcome following brain injury and treatment with an inhibitor of Nogo-A in association with an attenuated downregulation of hippocampal growth-associated protein-43 expression. J Neurosurg 107: 844–853. 17937233 10.3171/JNS-07/10/0844 PMC2366808

[36] Shen Y, Meiri K (2013) GAP-43 dependency defines distinct effects of netrin-1 on cortical and spinal neurite outgrowth and directional guidance. Int J Dev Neurosci 31: 11–20. 23085079 10.1016/j.ijdevneu.2012.10.006

[37] Laurino L (2005) PI3K activation by IGF-1 is essential for the regulation of membrane expansion at the nerve growth cone. Journal of Cell Science 118: 3653–3662. 16046480 10.1242/jcs.02490

[38] Virdee K, Xue L, Hemmings BA, Goemans C, Heumann R, et al (1999) Nerve growth factor-induced PKB/Akt activity is sustained by phosphoinositide 3-kinase dependent and independent signals in sympathetic neurons. Brain Res 837: 127–142. 10433995 10.1016/s0006-8993(99)01643-1

[39] Ma J, Zhang Z, Su Y, Kang L, Geng D, et al (2013) Magnetic stimulation modulates structural synaptic plasticity and regulates BDNF-TrkB signal pathway in cultured hippocampal neurons. Neurochem Int 62: 84–91. 23201339 10.1016/j.neuint.2012.11.010

